# Novel topical cyclosporine as adjuvant therapy for pyoderma gangrenosum: A case series

**DOI:** 10.1016/j.jdcr.2023.07.041

**Published:** 2023-08-20

**Authors:** Radhika Shah, Samantha Ouellette, Samavia Khan, Thu Truong, Amy Pappert, Cindy Wassef

**Affiliations:** a Department of Dermatology, Rutgers Robert Wood Johnson Medical School, Somerset, New Jersey; b Rutgers New Jersey Medical School, Newark, New Jersey

**Keywords:** dermatologic therapy, medical dermatology, pyoderma gangrenosum, topical cyclosporine

## Introduction

Pyoderma gangrenosum (PG) is characterized by rapidly progressing, painful cutaneous ulcers with undermined borders surrounded by erythema.^1^ Three major PG diagnostic criteria exist, Su et al criteria (2004), PARACELSUS score (2018), and Delphi Consensus Criteria (2018).^2^ PG is associated with a decreased quality of life and a nearly 3-fold increase in mortality.^3^ Current treatments for PG are based on a few, small studies. Systemic treatments include corticosteroids, cyclosporine, and antitumor necrosis factor agents.^4^ However, there is a risk of infections, hypertension, blood glucose abnormalities, renal dysfunction, and lipid abnormalities. Topical and intralesional corticosteroids confer a risk of developing skin atrophy, abnormal pigmentation, pathergy, and pain during injection administration. Due to the paucity of clinical data and the adverse effects of current treatments, identifying potential topical therapies remains an important area of research. This case series aims to demonstrate the safety and efficacy of topical cyclosporine as a treatment option for PG. Topical cyclosporine was prepared with 2 cyclosporine 100 mg capsules mixed in 98 mL of 100% vitamin E oil. This mixture was applied to PG ulcers once daily (Table I).

**Table I:** Previous and concurrent treatments, size of ulcer, and notable findings at follow-up visits for cases treated with topical cyclosporine

	Previous treatments	Concurrent treatments	Dosing of topical cyclosporine (CsA)	Size of ulcer	Follow-up
Case 1	Prednisone, topical clobetasol	Prednisone taper	Initial: 200 mg CsA/98 mL 100% Vit E once daily At 4 mo: 400 mg CsA/98 mL 100% Vit E once daily	23 cm × 15 cm	2 wk: decreased size, depth, and pain 3.5 mo: fully healed 4 mo: few satellite lesions appeared 5.5 mo: all but 1 satellite lesion resolved
Case 2	Adalimumab, intralesional triamcinolone, prednisone taper, infliximab, dapsone, clobetasol, methotrexate, and oral cyclosporine	Adalimumab, tramadol, gabapentin	200 mg CsA/98 mL 100% Vit E once daily	9 cm × 9 cm	3 wk: significant improvement in pain 8 wk: ulcer had healed into a pink granulated plaque with foci of 1 cm erosions
Case 3	Upaticinib, prednisone, intralesional triamcinolone, clobetasol paste mixed with ostomy powder, tacrolimus ointment, prednisone	Upacitinib, prednisone, intralesional triamcinolone	200 mg CsA/98 mL 100% Vit E once daily	2 cm × 1.5 cm	10 d: decreased pain, depth, size 6 wk: decreased in size (approx. 0.5 cm × 0.5 cm)
Case 4	Infliximab	Prednisone, vedolizumab	200 mg CsA/98 mL 100% Vit E once daily	19 cm × 19 cm	3 wk: decrease in pain, size (19 cm × 18.5 cm) 2 mon: decrease in pain, size (15 cm × 10 cm)
Case 5	Topical and oral corticosteroids, antibiotics, topical antifungals, prednisone taper, tacrolimus 0.1% ointment, triamcinolone 0.1% ointment	Prednisone taper, tacrolimus 0.1% ointment, triamcinolone 0.1% ointment	200 mg CsA/98 mL 100% Vit E once daily	4 ulcers: 4 cm, 2.5 cm, 5 cm, 2 cm	1 mo: decreased depth of ulcer 3 mo: decreased depth of ulcer
Case 6	Clobetasol, mupirocin, topical tacrolimus, and prednisone	Prednisone taper	200 mg CsA/98 mL 100% Vit E once daily	3 cm × 3 cm	4 wk: improved pain, depth 10 wk: decrease in size, depth (to 2 cm)
Case 7	Systemic steroids	None	200 mg CsA/98 mL 100% Vit E once daily	10 cm × 4 cm; 4.5 cm × 3 cm	5 wk: no improvement in ulcer

*Vit E*, Vitamin E.

## Cases

### Case 1

**Fig 1:**
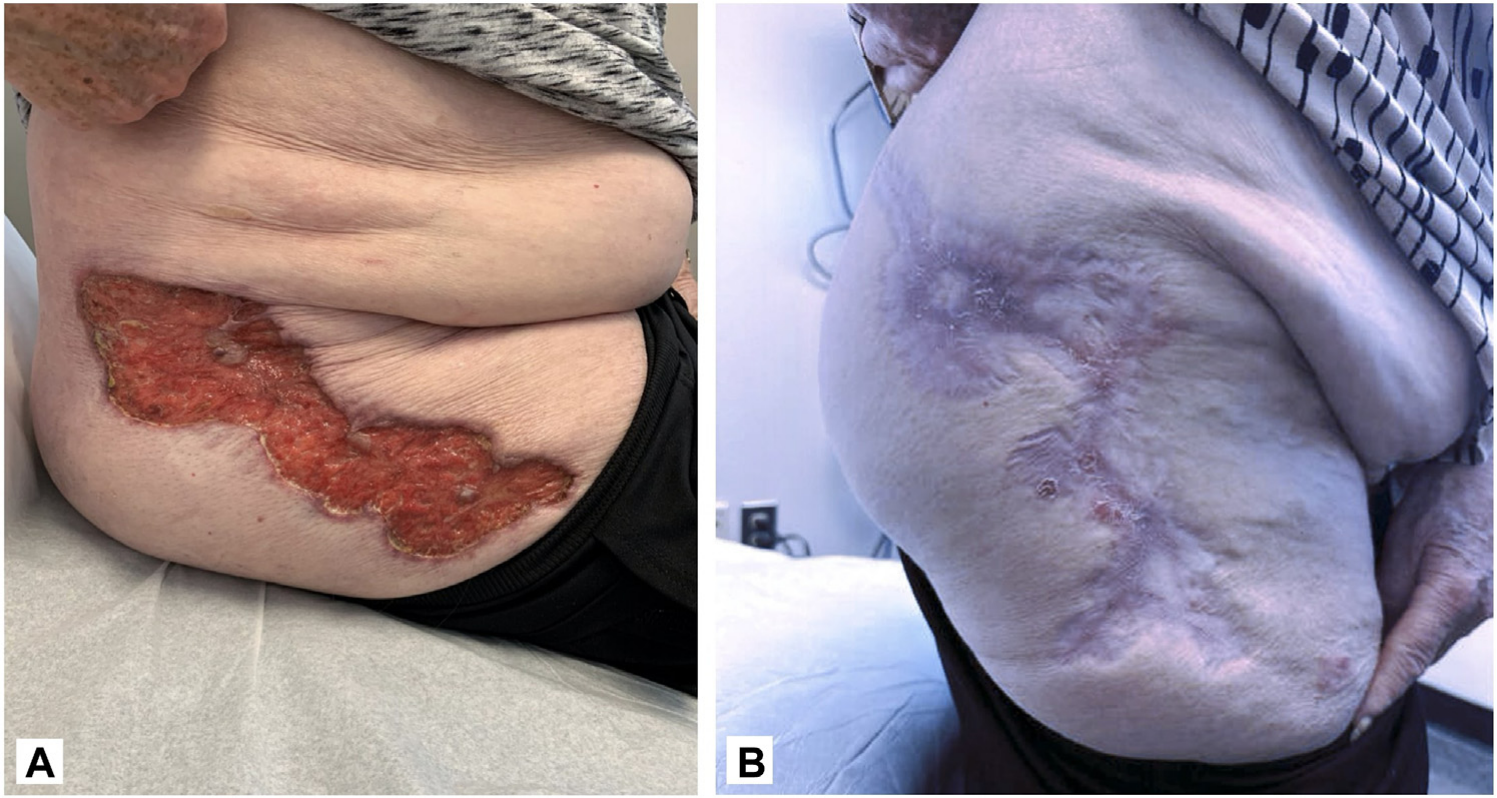
**A,** A 79-year-old female presenting at the initial visit with ulcer on the *right* lateral thigh measuring 23 cm × 15 cm. **B,** After 3.5 months of treatment with topical cyclosporine, the patient’s ulcer had fully healed.

A 79-year-old female with a history of colitis developed a recurrent ulcer on her right lateral thigh at the site of her hip replacement surgery 3 months prior to presentation. At the initial visit, a 23 × 15 cm ulcer with peripheral erythema (Fig 1, *A*) was noted that demonstrated a neutrophilic infiltrate on biopsy. Of the Delphi consensus PG criteria, 1 major criterion (biopsy) and 4 minor criteria (pathergy, history of inflammatory bowel disease, peripheral erythema at ulceration site, and exclusion of infection) were met, confirming the diagnosis. The patient was taking prednisone 60 mg and applying clobetasol daily without improvement. She reported that her pain was a 7/10 while on tramadol and gabapentin. The patient applied 200 mg CsA/98 mL 100% vitamin E once daily to the ulcer. Topical clobetasol was held and a prednisone taper was initiated. After 2 weeks of treatment, the patient’s ulcer decreased in both size and depth, and she reported significantly decreased pain, allowing her to discontinue tramadol. Prednisone was tapered off. By 3.5 months of treatment, her original lesion had fully healed without the patient experiencing any side effects from the topical cyclosporine (Fig 1, *B*). At 4 months, 3 satellite lesions appeared at the inferior margin of the original lesion, and her dose was increased to 400 mg CsA/98 mL 100% vitamin E once daily to the ulcer. At 5.5 months, all but one of the satellite lesions had resolved. Comprehensive metabolic panels (CMPs) at 4 and 7 months were significant only for minor changes to magnesium.

### Case 2

**Fig 2:**
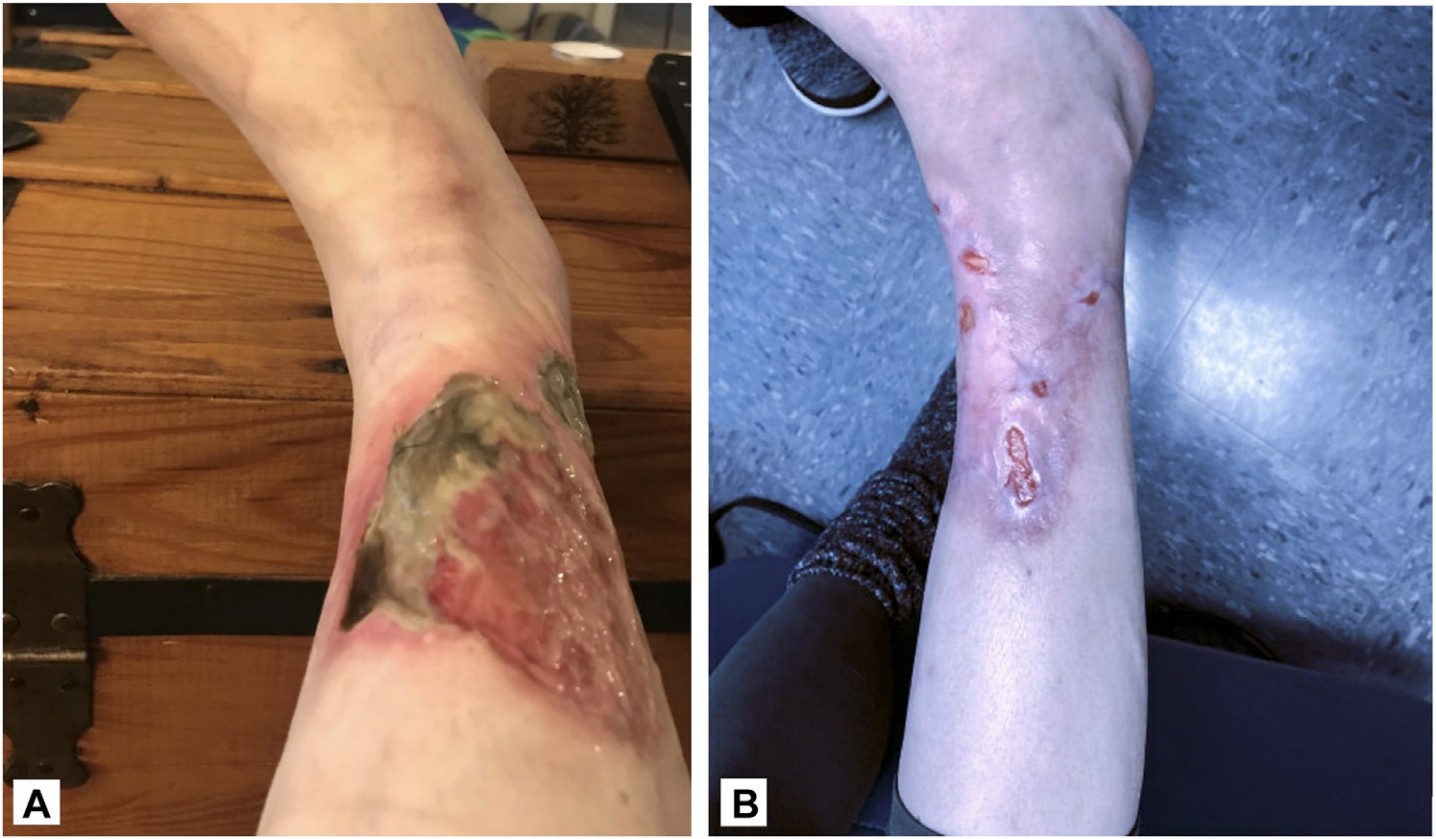
**A,** A 50-year-old female presenting at the initial visit with an ulcer measuring 9 cm × 9 cm on the *right* shin. **B,** After 4 weeks of treatment with topical cyclosporine, the patient’s ulcer had healed into a *pink* granulated plaque with focal erosions.

A 50-year-old female with a history of diabetes developed an ulcer on her right shin 2.5 years prior to presentation. A 9 × 9 ulcer was noted at the initial visit (Fig 2, *A*). Of the Su et al criteria, both major criteria (rapid progression of painful necrotic ulcer, exclusion of other causes of cutaneous ulceration) and 2 minor criteria (biopsy findings and clinical finding of cribriform scarring) were met, confirming a diagnosis of PG. The patient was not on any treatment. She reported 10/10 pain while on tramadol and gabapentin. Previously, the patient had tried adalimumab, intralesional triamcinolone injection, prednisone, infliximab, dapsone, clobetasol, methotrexate, and oral cyclosporine. Infliximab was discontinued due to intolerable nausea, vomiting, and fatigue, and oral cyclosporine caused an increase in creatinine, uric acid, and triglycerides. While adalimumab was well-tolerated, the ulcer continued to increase in size. The patient began 200 mg CsA/98 mL 100% vitamin E once daily to the ulcer and by 3 weeks, she had reported a significant improvement in pain and was able to discontinue tramadol. She also noted a decrease in ankle edema and ulcer depth, Repeat CMP at 4 weeks was within normal limits. By 8 weeks, the patient’s ulcer had healed into a pink granulated plaque with foci of 1 cm erosions (Fig 2, *B*). The patient did not report any side effects.

### Case 3

**Fig 3:**
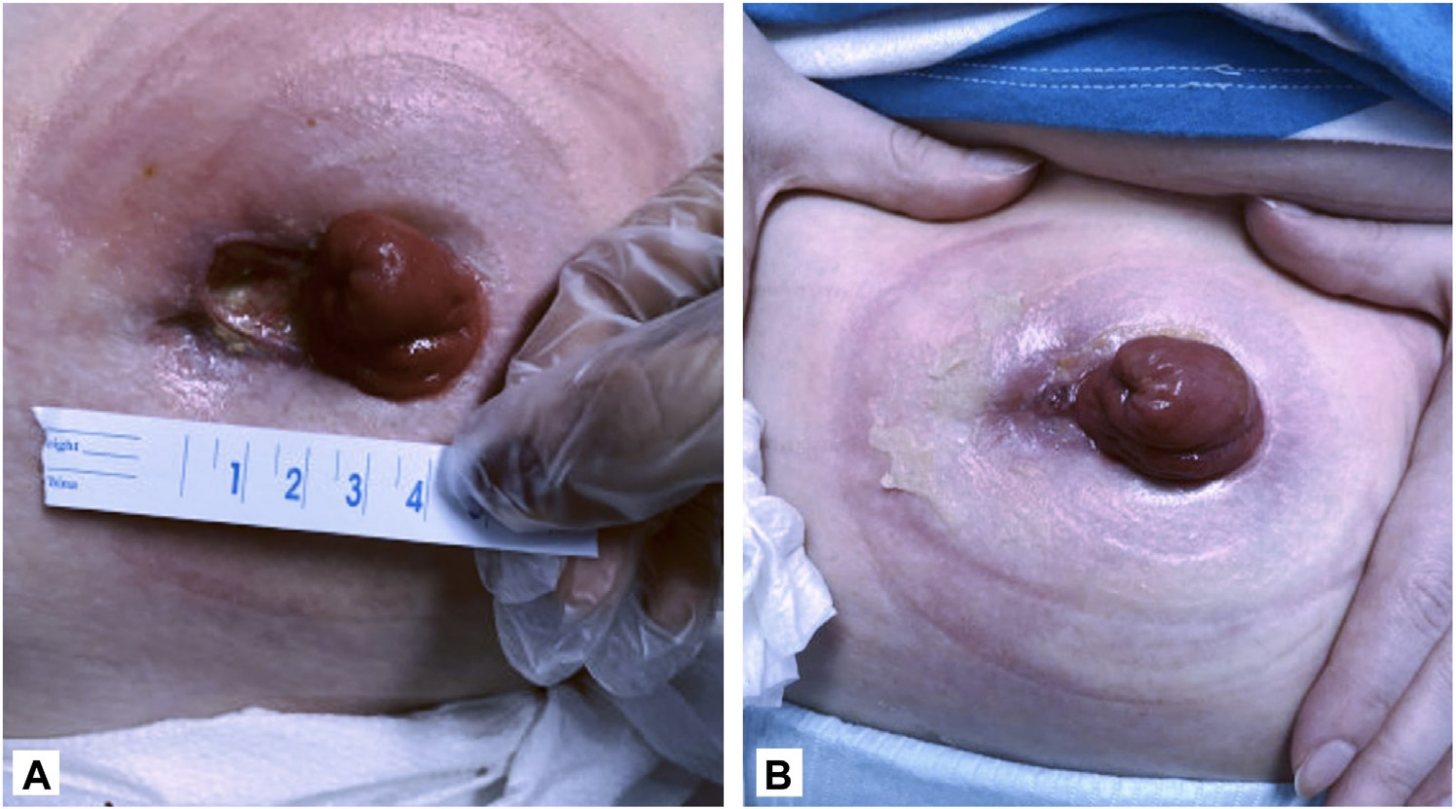
**A,** A 26-year-old female presenting to initial visit with an ulcer adjacent to her ostomy measuring 2 cm × 1.5 cm. **B,** After 6 weeks of treatment with topical cyclosporine, the patient’s ulcer measured 0.5 cm × 0.5 cm.

A 26-year-old female with a history of Crohn’s disease developed an ulcer on her abdomen adjacent to her ostomy site 2 years prior to presentation. At the initial visit, a 2 cm × 1.5 cm ulcer with peripheral erythema and undermined borders was noted (Fig 3, *A*). Of the Su et al criteria, both major criteria (rapid progression of painful necrotic ulcer and exclusion of other cutaneous causes of ulceration) and 2 minor criteria (history suggestive of pathergy, associated systemic diseases) were met, confirming a diagnosis of PG. The patient’s regimen included upadacitinib, prednisone, and intralesional triamcinolone 20 mg/mL injections to the site every 4 weeks. Trials of clobetasol paste mixed with ostomy powder, tacrolimus ointment, and prednisone were instituted without significant improvement. She endorsed osteoporosis, adrenal insufficiency, and weight gain from long-term steroid use. Additionally, she was on dapsone for 8 months which was discontinued after she developed hemolytic anemia, and adalimumab for 7 years which was discontinued after it did not improve her Crohn’s. The patient was then prescribed 200 mg CsA/98 mL 100% vitamin E and after 10 days of treatment, she reported improvement in pain, size, and depth. By 6 weeks, the ulcer had decreased in size to approximately 0.5 cm × 0.5 cm (Fig 3, *B*) and the patient did not require another intralesional triamcinolone injection since starting topical cyclosporine treatment. A CMP from 9 days of treatment showed an increase in cholesterol and triglycerides that was attributed to the patient’s upadacitinib. The patient denied any side effects.

### Case 4

**Fig 4:**
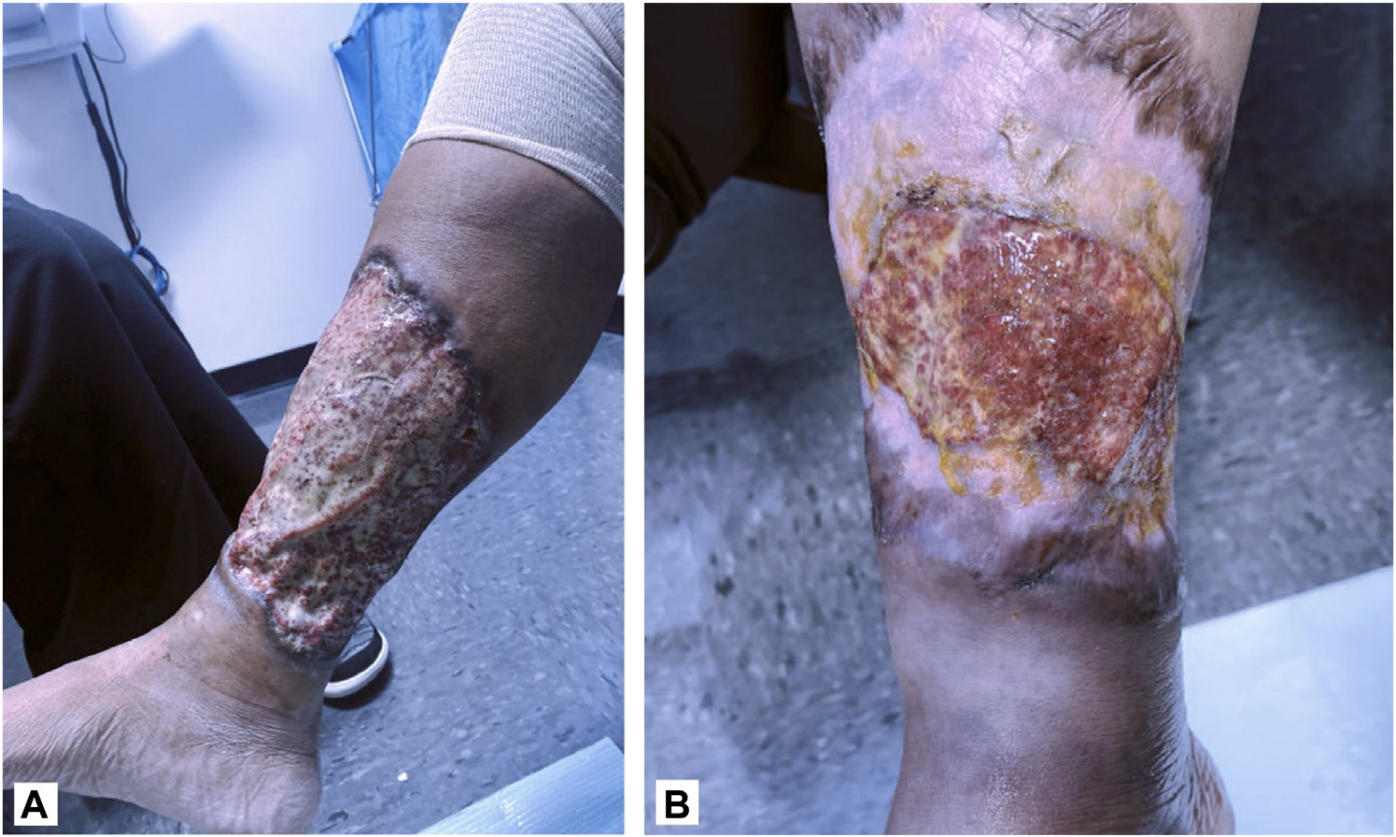
**A,** A 34-year-old female presenting to initial visit with an ulcer on her *right* shin measuring 19 cm × 19 cm. **B,** After 2 months of treatment with topical cyclosporine, the ulcer had decreased in size to 15 cm × 10 cm.

A 34-year-old female with a history of Crohn’s and sickle cell disease developed an ulcer on her right shin 2 months before presentation. At the initial visit, the ulcer measured 19 cm × 19 cm (Fig 4, *A*), and the pain was a 10/10. She was taking prednisone and vedolizumab. Of the Delphi consensus PG criteria, 1 major criterion (biopsy) and 4 minor criteria (history of inflammatory bowel disease, peripheral erythema and undermining at ulceration site, exclusion of infection, and multiple ulcerations with at least 1 developing on the anterior lower leg) were met, confirming the diagnosis. The patient was previously on infliximab, but it was discontinued after causing a sickle cell crisis. The patient started topical application of 200 mg CsA/98 mL 100% vitamin E daily to the ulcer. and after 3 weeks, she reported a decrease in pain from a 10/10 to a 7-8/10, and the ulcer had decreased in size to 19 cm × 18.5 cm. By 2 months, the ulcer had decreased in size to 15 cm × 10 cm (Fig 4, *B*) and reported her pain was 3-4/10. Patient reported no side effects and a complete blood count and CMP at 3 months showed no adverse changes.

### Case 5

**Fig 5:**
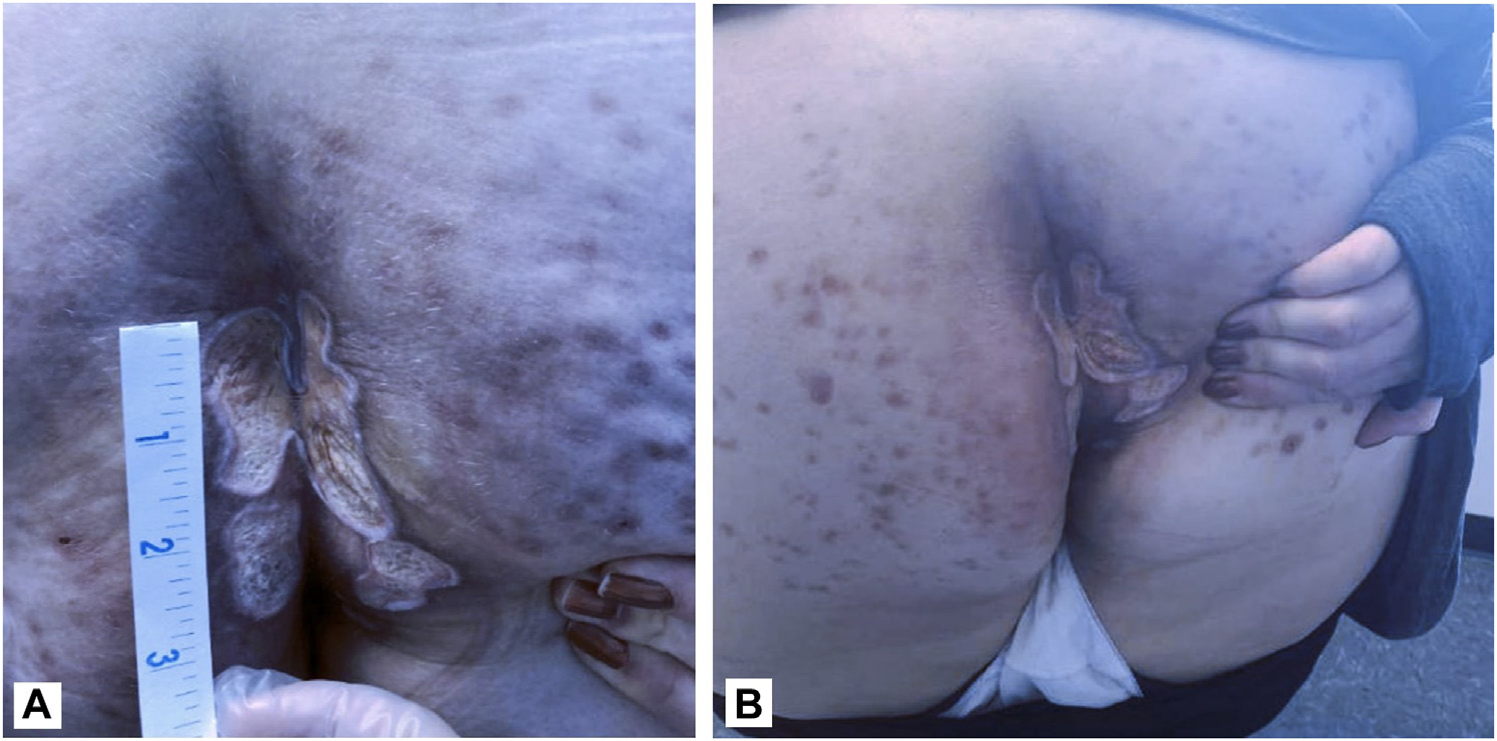
**A,** A 47-year-old female presenting to initial visit with ulcers on her *right* buttock measured 4 cm and 2.5 cm and the ulcers on her *left* buttock measured 5 cm and 2 cm. **B,** After 1 month of treatment with topical cyclosporine, the patient’s ulcers had decreased in depth.

A 47-year-old female with a history of hidradenitis suppurativa developed 4 ulcers in the intergluteal fold, 2 on the right and 2 on the left buttock, 2 years before presentation. Previous therapies included topical and oral corticosteroids, antibiotics, and topical antifungals, all of which were ineffective. At the initial visit, the ulcers on her right buttock measured 4 cm and 2.5 cm and the ulcers on her left buttock measured 5 cm and 2 cm (Fig 5). Of the Su et al criteria, both major criteria (rapid progression of painful necrotic ulcer and exclusion of other cutaneous causes) and 2 minor criteria (history of cribriform scarring and improvement with systemic steroids) were met, confirming a diagnosis of PG. At the time, the patient was on an 80 mg prednisone taper and applying tacrolimus 0.1% ointment and triamcinolone 0.1% ointment. The patient reported multiple side effects from prednisone including eye swelling, insomnia, high blood pressure, tremors, and weight gain. She was then prescribed 200 mg CsA/98 mL 100% vitamin E. to be applied once daily in addition to her daily regimen. Ulcer depth decreased at 1-month follow-up (Fig 5, *B*) with continued improvements in depth noted at 3 months. She reported symptomatic improvement with an overall increase in her quality of life. A complete blood count and CMP at 3 months of treatment showed no adverse changes from baseline. Despite the continued improvement of her ulcer without any reported side effects, the patient developed satellite lesions after discontinuing prednisone necessitating the addition of dapsone.

### Case 6

A 36-year-old male with a history of thrombocytopenia developed an ulcer on the left calf 7.5 months prior to presentation. At the initial visit, a 3 × 3 cm ulcer was noted that met major Su et al criteria (rapid progression of painful, necrolytic cutaneous ulcer and other cutaneous causes of ulceration excluded) and 2 minor criteria (cribriform scarring and histopathological findings of mixed inflammation), confirming a diagnosis of PG. The patient had previously failed clobetasol, mupirocin, topical tacrolimus, and prednisone. The patient was prescribed 200 mg CsA/98 mL 100% vitamin E. to be applied once daily. At the time of starting topical cyclosporine, the patient was on a prednisone taper at 40 mg daily. At 4 weeks of treatment, the ulcer had improved in pain and depth, and the patient was still on the prednisone taper at 5 mg daily. By 10 weeks of treatment, prednisone had been tapered off completely, and the ulcer had decreased in size and depth to a nontender crusted plaque measuring 2 cm.

### Case 7

A 71-year-old female with a history of hypertension, asthma, and cataracts developed 2 ulcers, 1 on her lateral right ankle and 1 on her medial right ankle 1 year before presentation. Of the Delphi consensus PG criteria, 1 major criterion (biopsy) and 4 minor criteria (peripheral erythema and undermining at ulceration site, exclusion of infection, multiple ulcerations—at least 1 developing on the anterior lower leg, and cribriform scars at healed sites) were met, confirming the diagnosis. At the initial visit, the ulcer on her lateral right ankle measured 10 cm × 4 cm and the ulcer on her medial right ankle measured 4.5 cm × 3 cm, and she was not on any treatment. Previously, the patient had the ulcers debrided without any improvements. The patient was prescribed 200 mg CsA/98 mL 100% vitamin E to be applied once daily as monotherapy, with a plan to start systemic steroids should the ulcers progress. At 5 weeks, the patient had no improvements with topical cyclosporine, so it was discontinued. Of note, the patient’s labs revealed a potential monoclonal gammopathy that may have interfered with the resolution of her ulcers. Additionally, the patient developed an irritant contact dermatitis at the site of application that resolved after discontinuation.

## Discussion

Six of 7 patients experienced an improvement in pain, depth, and size of their ulcers from topical cyclosporine. All 6 patients who responded to topical cyclosporine reported a noticeable decrease in pain by 4 weeks of treatment, with the earliest reported decrease in pain at 10 days of treatment. Two out of the 6 patients whose initial lesions responded to topical cyclosporine developed satellite lesions, necessitating the addition of another agent. Interestingly, one of the patients had a trial of prednisone prior to treatment with topical cyclosporine without ulcer improvement. However, the addition of prednisone to her topical cyclosporine regimen, in conjunction with an increase from 2% to 4% cyclosporine in vitamin E oil, led to the clearance of 2 out of 3 satellite lesions. While the second patient had an overlap of both prednisone and topical cyclosporine, satellite lesions developed only once prednisone was discontinued. This may indicate that prednisone and topical cyclosporine work synergistically on PG lesions. Only 1 patient reported a minor adverse effect of topical cyclosporine, an irritant contact dermatitis, which resolved upon discontinuation.

Two different topical cyclosporine formulations for PG have previously been reported. Azizan et al assessed the efficacy of topical cyclosporine prepared using the intravenous preparation of cyclosporine diluted 1:1 with distilled water.^5^ In this study, 3 of 4 patients had complete resolution of lesions after an average of 3.5 months of treatment. The patients did not endorse any side effects. Complete blood counts and biochemistry remained within normal limits and cyclosporine troughs remained at subtherapeutic levels throughout treatment. Urtea-Botero et al reported a 56-year-old woman with PG who was treated with topical cyclosporine eye drops (Restasis 0.05%) twice daily.^6^ Before starting topical cyclosporine, the patient was prescribed oral cyclosporine, which was discontinued after 4 weeks due to increased blood pressure. Additionally, the patient had tried prednisone; however, prednisone was discontinued due to hyperglycemia. With topical cyclosporine treatment, the patient experienced a reduction in pain, and within 8 weeks, the PG ulcer had healed.

In this study, we prescribed modified cyclosporine (Neoral) 100 mg capsules and instructed to mix the contents of 2 capsules into 98 mL of 100% vitamin E oil. Vitamin E oil was chosen due to its fat-solubility, accessibility, and intrinsic antiinflammatory properties.^7^ Compared to the prior 2 studies, our treatment modality is more cost-effective and can be made in larger quantities based on ulcer size. Additionally, concentration can be easily modified while maintaining efficacy and avoiding systemic side effects. Setbacks to using IV formulation of cyclosporine include obtaining the medication as well as cost of treatment. Topical cyclosporine eye drops are often not covered by the patient’s insurance and are only available in 0.05% 5 mL size compared to 2% to 4% used in our study.

## Conclusion

PG is a debilitating condition that reduces patients’ quality of life and can increase mortality. While systemic and topical treatments are available, these agents often lack efficacy, with adverse effects and cost barriers associated with systemic agents. The topical cyclosporine formulation in this study demonstrates a cost-effective, seemingly efficacious agent with minimal associated adverse effects that can either be a monotherapy or an adjuvant to expedite ulcer resolution.

## Conflicts of interest

None disclosed.
